# Spectrum Bias and Individual Strengths of SARS-CoV-2 Serological Tests—A Population-Based Evaluation

**DOI:** 10.3390/diagnostics11101843

**Published:** 2021-10-06

**Authors:** Sebastian Einhauser, David Peterhoff, Hans Helmut Niller, Stephanie Beileke, Felix Günther, Philipp Steininger, Ralph Burkhardt, Iris M. Heid, Annette B. Pfahlberg, Klaus Überla, Olaf Gefeller, Ralf Wagner

**Affiliations:** 1 Institute of Medical Microbiology and Hygiene, Molecular Microbiology (Virology), University of Regensburg, Franz-Josef-Strauß-Allee 11, 93053 Regensburg, Germany; sebastian.einhauser@klinik.uni-regensburg.de (S.E.); david.peterhoff@klinik.uni-regensburg.de (D.P.); Hans-Helmut.Niller@klinik.uni-regensburg.de (H.H.N.); 2 Institute of Clinical Microbiology and Hygiene, University Hospital Regensburg, Franz-Josef-Strauß-Allee 11, 93053 Regensburg, Germany; 3 Institute of Clinical and Molecular Virology, University Hospital Erlangen, Friedrich-Alexander Universität Erlangen-Nürnberg, Schlossgarten 4, 91054 Erlangen, Germany; Stephanie.Beileke@uk-erlangen.de (S.B.); philipp.steininger@uk-erlangen.de (P.S.); klaus.ueberla@fau.de (K.Ü.); 4 Department of Statistics, Statistical Consulting Unit StaBLab, LMU Munich, Geschwister-Scholl-Platz 1, 80539 Munich, Germany; felix.guenther@stat.uni-muenchen.de; 5 Department of Genetic Epidemiology, University of Regensburg, Franz-Josef-Strauß-Allee 11, 93053 Regensburg, Germany; Iris.heid@ur.de; 6 Institute of Clinical Chemistry and Laboratory Medicine, University Hospital Regensburg, Franz-Josef-Strauß-Allee 11, 93053 Regensburg, Germany; ralph.burkhardt@klinik.uni-regensburg.de; 7 Department of Medical Informatics, Biometry and Epidemiology, Friedrich-Alexander Universität Erlangen-Nürnberg (FAU), Waldstr. 6, 91054 Erlangen, Germany; annette.pfahlberg@fau.de

**Keywords:** SARS-CoV-2, ELISA, ECLIA, neutralization, spectrum bias, serology, test accuracy, sensitivity, specificity

## Abstract

Antibody testing for determining the SARS-CoV-2 serostatus was rapidly introduced in early 2020 and since then has been gaining special emphasis regarding correlates of protection. With limited access to representative samples with known SARS-CoV-2 infection status during the initial period of test development and validation, spectrum bias has to be considered when moving from a “test establishment setting” to population-based settings, in which antibody testing is currently implemented. To provide insights into the presence and magnitude of spectrum bias and to estimate performance measures of antibody testing in a population-based environment, we compared SARS-CoV-2 neutralization to a battery of serological tests and latent class analyses (LCA) in a subgroup (*n* = 856) of the larger population based TiKoCo-19 cohort (*n* = 4185). Regarding spectrum bias, we could proof notable differences in test sensitivities and specificities when moving to a population-based setting, with larger effects visible in earlier registered tests. While in the population-based setting the two Roche ELECSYS anti-SARS-CoV-2 tests outperformed every other test and even LCA regarding sensitivity and specificity in dichotomous testing, they didn’t provide satisfying quantitative correlation with neutralization capacity. In contrast, our in-house anti SARS-CoV-2-Spike receptor binding domain (RBD) IgG-ELISA (enzyme-linked-immunosorbant assay) though inferior in dichotomous testing, provided satisfactory quantitative correlation and may thus represent a better correlate of protection. In summary, all tests, led by the two Roche tests, provided sufficient accuracy for dichotomous identification of neutralizing sera, with increasing spectrum bias visible in earlier registered tests, while the majority of tests, except the RBD-ELISA, didn’t provide satisfactory quantitative correlations.

## 1. Introduction

Coinciding with the onset of the SARS-CoV2 pandemic in early 2020 there was an urgent and strong quest for serological tests addressing a number of different needs, for example, to determine the number of undetected infections enabling the calculation of infection fatality ratios, to survey virus spread at the population level and also to serve immediate medical needs, namely to qualify convalescent plasma for treatment of severely diseased patients. Alongside with others and in order to meet such needs, we established and validated a SARS-CoV-2 ELISA for the detection and quantification of antibodies targeting the receptor binding domain (RBD) of the spike (S) protein for in-house diagnostic use. The assay itself was validated, using sera from the pre-pandemic era to determine test specificity as well as available positive sera, which at that time were derived from PCR positive, symptomatic and partially hospitalized COVID-19 patients to calculate test sensitivity [1]. A similar strategy, though using the SARS-CoV-2 nucleoprotein (N) instead of the spike protein for antibody detection, was applied like for many other now commercially available SARS-CoV-2 antibody tests–for validation and approval of the Roche COBAS/ELECSYS SARS-CoV-2-N (nucleoprotein) platform, where 204 samples of 69 PCR-positive patients were tested at various time points, and 5272 pre-December 2019 samples were used to test for specificity [2]. Though various studies and meta-analyses have been carried out comparing different serological tests during the pandemic [3,4,5], most of these studies used predefined clinical samples relying on pre-COVID-19 and symptomatic/PCR-verified cohorts or other, non-population based sera or convenience samples to derive performance metrics for test accuracy.

Despite being methodologically correct from a laboratory and experimental perspective, such comparative studies may harbor the risk of a potential “spectrum bias”, a phenomenon already described by Ransohoff and Feinstein more than 40 years ago when scrutinizing the evaluation process of the accuracy of diagnostic tests [6]. After repeated observations of failure of initially promising new diagnostic tests when introduced in clinical practice, this discrepancy could be attributed to differences in the composition of samples used for test validation and the final target population. Although in statistical theory measures like sensitivity and specificity are population-independent test-specific constants, in biological reality these measures vary between test populations, as often the condition to be detected is not inherently dichotomous [7,8]. Thus, it is crucial to use an appropriate test population, resembling the setting in which the diagnostic test is used in later practice, when evaluating diagnostic tests. Therefore, population-based studies are critically important to determine and compare performance measures like sensitivity and specificity of SARS-CoV-2 antibody assays.

With an increasing number of individuals recovered from COVID-19 and the success of vaccination campaigns, the initial quest for tests accurately determining the serostatus in a dichotomous fashion is currently being complemented by a growing demand for a simple and quantitative surrogate, which can predict protection from infection or disease in convalescent and vaccinees [9]. To avoid any spectrum bias, such surrogates should be validated based on a representative and properly powered set of samples from the population.

The objective of this study therefore was to analyze and to compare different SARS-CoV-2 serological tests in a population-based setting following STARD (Studies of diagnostic accuracy) guidelines [10,11]. Furthermore, we opted to determine the quantitative agreement of the obtained test results with virus neutralization representing the currently best predictive immunological correlate for protection. For didactical reasons we also aimed to highlight the impact of potential spectrum bias on diagnostic performance measures.

## 2. Materials and Methods

### 2.1. Study Design and Participants

For this retrospective analysis we used a subgroup of the well described population-based TiKoCo-19 study cohort [10]. In brief, a representative sample of 6608 residents of Tirschenreuth aged 14 years and older, selected by means of a sex- and municipality-stratified random sample, were invited and 4203 (64.27%) fully participated in the study by filling out a questionnaire and giving blood samples. Three independent antibody tests were applied (complete data from 4185 participants) and the “true” serostatus was predicted by latent class analysis, to finally estimate key epidemiological parameters like seroprevalence and infection fatality ratio [10].

Out of these 4185 participants, 432 tested positive for SARS-CoV-2 antibodies in at least one of the three antibody tests and thus were defined as “any-test-positive”. As neutralization testing remains time and work intensive, 430 of these, which yielded sufficient material for further testing were analyzed for their age and sex distribution and equivalent number (426) of age- and sex-matched samples were then randomly selected from the remaining “all-tests-negative” participants. Being aware that a reduction of the SARS-CoV-2 antibody negative samples while keeping the positive population constant will induce a bias in the subgroup analysis, we estimated all diagnostic performance measures after applying a reweighting procedure to yield a subgroup for the analysis resembling the full TiKoCo-19 study cohort (*n* = 4185). This weighting technique uses weights inverse proportional to the probability of being included in the subgroup and, in general, will avoid the bias in the estimation of diagnostic performance parameters otherwise encountered [12]. Thus, a final population-based subgroup of *n* = 856 was defined with the following characteristics: male (*n*; %) = 410; 48; age (median; range) = 52; 14–100.

Of note, by the nature of the study design, it was impossible to assess exact time intervals between SARS-CoV-2 infection and taking blood samples, while epidemiological data from Robert Koch Institute (RKI) strongly suggests that nearly all infections during that first wave in the county of Tirschenreuth happened 2–4 months prior to the 1st day of taking blood samples allowing for seroconversion [13]. No timepoint differences have to be assumed between the tests, as all analyses were performed from the same blood sample.

### 2.2. “Wetlab”-Methods

#### 2.2.1. Independence of Testing

All serological tests were performed by independent experimentators in a randomized and pseudonymized manner, while the experimentator were blinded for any other test result during testing. Neutralization and Elecsys Anti-SARS-CoV-2 Spike test (Roche Diagnostics GmbH, Penzberg, Germany) was separately performed on the “any-test-seropositive/negative” (in-house anti SARS-CoV-2-Spike-RBD IgG-ELISA; YHLO anti-SARS CoV-2 test (Shenzhen Yhlo Biotech Co. Ltd., Shenzen, China) and Elecsys Anti-SARS-CoV-2 Nucleoprotein test (Roche Diagnostics GmbH, Penzberg, Germany)) subgroup (Table 1; detailed test description Section 2.2.2, Section 2.2.3, Section 2.2.4, Section 2.2.5, Section 2.2.6). Experimentors were neither aware which specific test delivered seropositive results nor did they have insight into any quantitative values.

#### 2.2.2. SARS-CoV-2 in-House S_RBD_-ELISA

Our validated in-house ELISA detecting IgG (or IgA or IgM) antibody responses to the SARS-CoV-2 spike protein’s receptor binding domain (RBD) was performed as described earlier [1]. Results from the different immunoglobulin classes were either used on their own (ELISA_G (IgG); ELISA_A (IgA); ELISA_M (IgM)) or combined to ELISA_GAM using the “believe-the-positive” rule, i.e., only if the results of all three immunoglobulin classes were negative, ELISA_GAM was defined negative. Cutoff values were chosen as published earlier. Test characteristics are shown in Table 1.

#### 2.2.3. Roche SARS-CoV-2 ELECSYS S Antibody Test

The Elecsys Anti-SARS-CoV-2 S test (Roche Diagnostics GmbH) [14] detecting complete Ig directed to Spike-(S) protein receptor-binding-domain (RBD) was operated on the COBAS pro e 801 module according to the manufacturer’s recommendations. Cutoff values were chosen as specified by the manufacturer. Test characteristics are shown in Table 1.

#### 2.2.4. Roche SARS-CoV-2 ELECSYS N Antibody Test

The Elecsys Anti-SARS-CoV-2 test (Roche Diagnostics GmbH, Penzberg, Germany) [15] detecting nucleoprotein-(N)-directed complete Ig was operated on the COBAS pro e 801 module according to the manufacturer’s recommendations. Cutoff values were chosen as specified by the manufacturer. Test characteristics are shown in Table 1.

#### 2.2.5. YHLO SARS CoV-2 Test

The YHLO SARS CoV-2 test (Shenzhen Yhlo Biotech Co. Ltd., Shenzen, China) detects IgG antibodies to the N- and S-protein and was performed on the iFlash 1800 (Shenzhen Yhlo Biotech Co. Ltd., Shenzen, China) according to the manufacturer’s recommendations. Cutoff values were chosen as specified by the manufacturer. Test characteristics are shown in Table 1.

#### 2.2.6. SARS-CoV-2 Neutralization Test

SARS-CoV-2 neutralization capacity was evaluated using the Vesicular Stomatitis Virus (VSV–Δ G*FLuc) pseudotyped with SARS-CoV-2-Spike-ΔER [16], which is an established and widely used surrogate for WT SARS-CoV-2 neutralization [17,18] (Supplemental Figure S1) while providing enhanced (bio)safety. Pseudoviral titers were determined by limited dilution and fluorescence microscopy. For all samples, an inoculum of 25,000 ffu was neutralized with a 2-fold serum dilution series starting at 1/20 dilutions in triplicates for 1 h, and luciferase activity was determined 20 h post infection of HEK293T-ACE2 + -cells using BrightGlo (Promega Corp, Madison, WI, USA). IC50 values (50% maximal inhibitory concentration) were calculated using the algorithm: ‘log (inhibitor) vs. normalized response’ in GraphPad Prism 8 software (GraphPad Software, San Diego, CA, USA). For dichotomous analysis, values above IC50 ≥ 20 were defined positive.

### 2.3. Statistical Analysis

#### 2.3.1. Dichotomized Data on Serostatus

Data from antibody testing were dichotomized following guidance from the manufacturers’ manuals to define serostatus and thereby distinguishing seropositive from seronegative samples for each antibody test separately. Agreement on serostatus between the different antibody testing procedures was quantitatively assessed by estimates of Cohen’s kappa, a measure of chance-corrected agreement ranging between 0 and 1 that avoids the pitfall of simply quantifying total agreement percentages [19]. Estimates of Cohen’s kappa are accompanied by 95% confidence intervals (CIs) to depict the precision of the point estimates.

The diagnostic performance of single antibody tests is evaluated based on the dichotomized results of the neutralization assay. In addition to quantifying agreement using Cohen’s kappa we also estimated sensitivity, specificity and the Youden index combining these two measures of diagnostic performance taking the neutralization assay as the gold standard to define true serostatus. In addition, we report measures of the predictive performance of test results on the population level, namely the positive predictive value (PPV) and the negative predictive value (NPV). Estimates of all these measures are accompanied by 95%-CIs derived by Wilson’s method [20].

In a further step we also analyzed the diagnostic performance of model-predicted serostatus derived from various latent class models that use the information from all or subsets of the single antibody tests. Latent class analysis (LCA), a classical modeling approach for discrete data developed more 70 years ago by Lazarsfeld [21], has increasingly been applied during recent decades in the context of infectious disease data when a number of different diagnostic tests but no established gold standard are available [22]. In general, LCA identifies a set of discrete, mutually exclusive latent (i.e., unobserved) classes based on the observed pattern of a set of categorical variables. The basic idea of LCA in this setting is to treat the unobservable true serostatus as being equivalent to two latent classes (seropositive vs. seronegative) and to relate the observed pattern of antibody test results to it via a statistical model. The model derived from LCA provides an objective way of classifying the partially contradictory pattern of results from antibody testing into the two groups of seropositives and seronegatives. We estimated agreement between LCA classification and dichotomized results of neutralization assays using Cohen’s kappa and also sensitivity, specificity and the Youden index taking the neutralization assay as the gold standard for serostatus.

#### 2.3.2. Raw Data on Seropositivity

We analyzed the raw (undichotomized) data of the antibody tests to elucidate the correlation structure of the quantitative readouts between different antibody tests. We restricted this correlation analysis to the subsample of seropositives that have been classified seropositive by all four antibody tests as inclusion of seronegatives would bias this analysis. We eliminated three samples where the quantitative value of an antibody test exceeded the upper cutoff defined by the manufacturer leaving a remaining subgroup of 310 seropositives contributing to this analysis. Raw data of the COBAS_S, COBAS_N and YHLO were log-transformed, raw data of the ELISA remained untransformed. Estimates of Pearson’s correlation coefficient (accompanied by 95%-CIs) are reported to quantify the strength of a linear association between quantitative test results. Additionally, estimates of Spearman’s rank correlation coefficient (accompanied by 95%-CIs) are shown to give a quantitative impression of the strength of a more general monotonous association between test results.

#### 2.3.3. Illustration of Spectrum Bias

Our data set for previous analyses comprised a randomly selected (weighted) population-based study group. Thus, the evaluation of the diagnostic performance of a serological testing using different specific procedures avoids spectrum bias that is inherent in many other settings. To illustrate the effect of spectrum bias on measures of diagnostic performance (sensitivity, specificity, Youden index, PPV and NPV) we generated a selected subgroup for an alternative analysis. The data set for the illustration is constructed by eliminating all 241 observations where the neutralization assay yielded values above 1 but below 100. The remaining sample of 615 observations comprises only clear-cut seronegatives and seropositives and serves as an instructive example of the effects of spectrum bias. We withstood the temptation to vary the exclusion criteria with respect to the width of the interval of values to be excluded in an effort to find a stark example, we only performed our analysis once with the pre-specified eligibility criteria stated above and report the corresponding results.

#### 2.3.4. Software and Tools

The statistical software SAS version 9.4 (SAS Institute Inc., Cary, NC, USA) was used for all computations, and LCA modeling was performed by employing a SAS extension from the Penn State University [23].

## 3. Results

### 3.1. Agreement between Dichotomized Serological Tests

We compared the dichotomized (seropositive vs. seronegative) results of the different serological tests to assess agreement regarding the identification of the serostatus. Though targeting antibodies to a different antigen (Table 1) but using the same test system we found a nearly perfect agreement between the two COBAS tests (κ = 0.96), while both, the IgG ELISA and the YHLO provided very good agreement with both COBAS tests (each κ ~ 0.9) and the YHLO and the ELISA showed a similar degree of agreement (κ = 0.87).

Expectedly, as could be anticipated from earlier reports analyzing the occurrence and durability of IgA and IgG antibody isotypes, the IgA and IgM ELISA on their own showed only weak agreement with every other test (each κ ≤ 0.21), respectively. In an attempt to improve the IgG ELISA’s performance, we combined the three ELISAs (G, A, M) with an OR-function, rendering every participant with at least one positive result in any test as seropositive. Though the results were strongly reliant on the IgG ELISA (κ = 0.93), the combination did not improve but rather decreased the overlap with the other tests (Table 2).

### 3.2. Relationship between Dichotomized Serological Tests and Neutralization

Next, we compared the test results obtained for the individual binding antibody assays with the presence or absence of neutralizing antibodies. The COBAS_S test, designed to detect and quantify antibodies directed towards the ACE-2 receptor-binding-domain (RBD) of the Spike (S) protein, was able to identify neutralizing sera on a near to perfect level as expressed by the Youden Index (J) (J = 0.97) with high sensitivity (97.2%) and specificity (99.7%).

The COBAS_N test scored second best on both parameters resulting in a Youden-Index of 0.94. This was not necessarily expected as *n* is not presented on the virion’s surface and most likely only S-protein directed antibodies exhibit neutralization capacity. The YHLO and the ELISA test showed comparable overall performance (J = 0.85 and 0.88, respectively), whereas the ELISA demonstrated a notable higher sensitivity (89.2% vs. 85.3%), and the YHLO exhibited slightly enhanced specificity (99.4% vs. 98.9%). While the IgA and IgM ELISA only showed minimal suitability for this issue (both J < 0.2), the combined ELISA data (ELISA_GAM J = 0.87) increased test sensitivity to 92.2%. This was, however, on high cost of assay specificity and couldn’t further improve the already good ELISA_G estimates (J = 0.88) (Table 3).

### 3.3. Illustration of the Effect of Spectrum Bias on Diagnostic Performance Measure

Since previous studies suggest that hospitalized COVID19 patients yield higher titers of neutralizing antibodies [24], while pre-2019 sera, by definition should not and also in reality have not been found to neutralize SARS-CoV-2 so far, we defined an artificial “establishment sample setting” within our study sample, by defining a subgroup comprising only including participants with a very definite neutralization titer of either IC_50_ < 1 or >100 (*n* = 615) and normalized those results back to the full cohort (*n* = 4185) for proper comparability.

To illustrate the potential impact of a spectrum bias we compared this “artificial establishment cohort” to the real population-based findings. By this procedure, we found clearly enhanced performance of every test, especially regarding test sensitivity. The highest impact was determined for the YHLO, which gained nearly 10% sensitivity compared to the population-based setting, followed by the ELISA_G (∆(sens) = 9%). The COBAS_N test gained approximately 7% in sensitivity, with only minor gains in specificity, impressively even reaching 100% sensitivity, while the COBAS_S still gained 2% sensitivity, but nearly no specificity (Table 3).

Interestingly, this pattern resembles the time of licensing of the tests, with the biggest gains to be recorded for the earlier licensed tests. Overall, in this artificial setting, all test determinants closely resembled the manufacturer’s information (Table 1) regarding sensitivity and specificity.

Those findings are also reflected by PPV and NPV, respectively. While the two COBAS tests exhibit superior values even at the rather low seroprevalence of 8.6%, YHLO and ELISA show slightly lower, but still solid PPVs. As expected from above reported test parameters, spectrum bias is visible, especially in PPVs, for all tests (Table 3, Supplemental Figure S2).

### 3.4. Relationship between Serostatus Predicted by Latent Class Modelling and Neutralization Results

Lacking a suitable gold standard to determine seroprevalence in our population based TiKoCo-19 baseline study, we applied latent class modelling by combining test results derived from the COBAS_N test with the YHLO assay and our in-house IgG ELISA as the best approximation to determine true serostatus. Using our neutralization data as functional reference, we were asking: (i) how the LCA performs regarding the identification of neutralizing sera and (ii) whether the performance of the LCA based on initial criteria can be further improved by adding data from additional serological tests (e.g., COBAS_S; ELISA IgM (ELISA_M) and IgA (ELISA_A)) to the LCA analysis. The first LCA-model (LCA1) mimicked the model used in the TiKoCo-19 baseline study and provides a very good prediction of the presence of neutralizing antibodies (J = 0.913). Whereas the inclusion of ELISA_A and M results per se did not influence the model’s parameters at all (data not shown), the replacement of ELISA_G by ELISA_GAM was able to minimally improve the model’s performance (LCA2; J = 0.915).

As the Roche COBAS_S wasn’t available in June 2020 when the baseline study was conducted, we couldn’t include its results at the time of the TiKoCo-19 baseline study. Whilst now we found a noticeable improvement of the baseline model (LCA1) when including this test in the latent class modelling of the subgroup (LCA3) (J = 0.946). Again, as for the above, including the IgA and IgM ELISA data in any combination couldn’t further improve the model at all (LCA4&4b, J = 0.946) (Table 4). This is expected since the IgM ELISA due to early occurrence of IgM antibodies is only sensitive in an early time-interval after infection and thus not suited to predict general seropositivity [25].

Interestingly, comparing the best-in-class LCA model (Table 4, LCA3) with the best-in-class individual test (Table 3, COBAS_S), the performance regarding assay sensitivity and specificity turned out to be similar. In conclusion, the COBAS_S test provided the most feasible surrogate for the dichotomous antibody detection in neutralizing sera. Prediction of serostatus by latent class modelling incorporating further serological tests could not improve the performance of the single COBAS_S test.

### 3.5. Correlation of Quantitative Serological Test Results and Neutralization

In view of globally ongoing vaccination campaigns, emerging variants of concern and the desire of our societies, opinion leaders in economy and politics to relief restrictions and return “back to normal”, there is an increasing quest for feasible surrogates to predict protection from infection or disease. Whilst the levels of neutralizing antibodies currently represent the best correlate of protection, such assays are cumbersome to conduct, low to medium throughput, time consuming and expensive. The quantification of binding antibodies might represent a feasible surrogate, provided the quantitative date show a sufficiently good correlation.

Herein, we used Pearson’s correlation coefficients to evaluate quantitatively the association between test results for binding antibodies and neutralization, respectively. This analysis proved a satisfactory correlation between the two tests designed to quantify the levels of antibodies binding to the ACE-2 receptor-binding domain (RBD) of the S protein (ELISA_G; COBAS_S; R = 0.74) as well as for the two N-based readouts (COBAS_N; YHLO; R = 0.77). As expected, since they test for different antigen-targets, we couldn’t find a sufficient correlation between the S and the N-based tests, though the YHLO-test claims to include spike antibodies as a target, which results in a slightly better correlation coefficient to the ELISA_G and the COBAS_S as compared to the COBAS_N (R (YHLO-ELISA) = 0.56; other R < 0.5).

**Figure 1:**
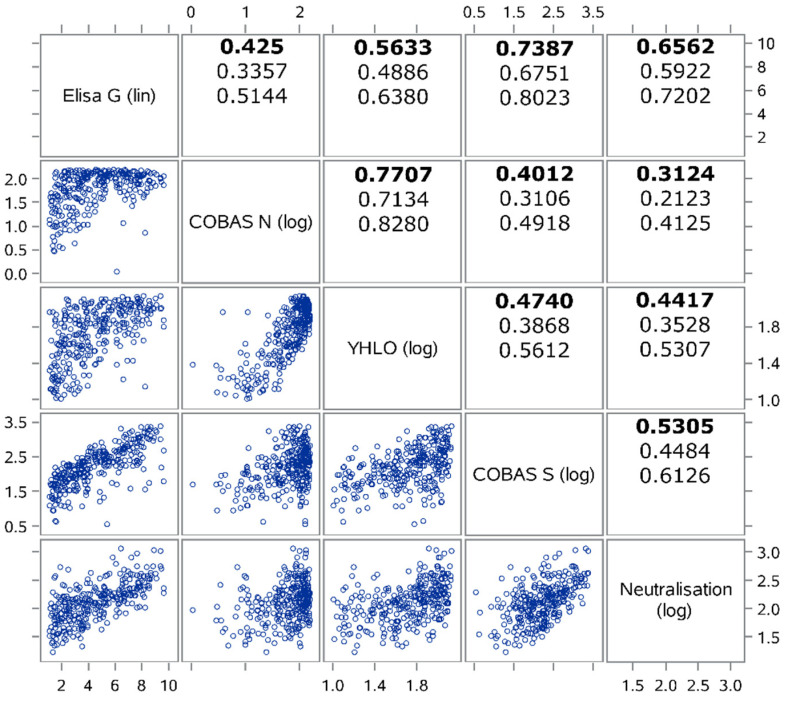
Scatterplot matrix of correlation between the different positive test results using logarithmic results for ECLIA based tests and neutralization. Also shown are Pearson correlation coefficients (bold type, 95%-CIs in the two rows below) to quantify the magnitude of linear association between antibody tests and neutralization within the subgroup (*n* = 310) where all tests (ELISA_G, COBAS_N, COBA_S; YHLO; neutralization) were positive. (Further experimental inclusion constraints: COBAS_S ≤ 2500; ELISA_G ≤ 15; Neutralization IC_50_ ≤ 2560).

Comparing the levels of binding antibodies quantified by the various serological tests to virus neutralization (IC_50_), we found a satisfactory correlation of the S-RBD-ELISA with neutralizing antibodies (R = 0.65), followed by an only moderate correlation of the COBAS_S (R = 0.53), while both, YHLO and COBAS_N showed only minor quantitative correlation to the determined IC50 values (R < 0.5) (Figure 1). Off note, the COBAS_N is not registered for quantitative readout and measurements often provided maximum reads, which may have negatively impacted correlations calculated for that test with other assays. We further investigated for non-linear monotonic associations, by using Spearman correlations, which overall didn’t lead to different results (Supplemental Table S1).

## 4. Discussion

While PCR testing is still the reference method to identify acute COVID-19 cases [26], serological testing is widely used in epidemiological studies to gather information on the dynamics of the pandemic [27], regarding the impact of political and health care measures, the number of undetected infections, the infection fatality ratio [10] or even identification of high antibody-titer convalescent sera for the treatment of severe COVID cases [3,28]. Manufacturers’ information on test performance relies on the composition of the test population that was available at the time of development and validation of these tests. While pre-pandemic sera were used to determine test specificity, test sensitivity was presumably evaluated based on-at that time-available samples of symptomatic and in part hospitalized COVID-19 cases with positive PCR tests largely missing, e.g., asymptomatic infected. Based on such a composition of the test population in this “test establishment and validation setting”–which is fundamentally different from a population-based setting in which these serological tests are later on applied any statement on test performance is prone to spectrum bias. Our results demonstrate that spectrum bias is not only a theoretical threat to the validity of manufacturers’ initial claims on test performance. An unbiased assessment of the properties of serological tests in identifying neutralizing capacity in a population-based setting yields slightly less perfect but still remarkably good test performance of these assays.

A new scope of application of serological testing is gaining increasing importance. With the ongoing SARS-CoV-2 vaccine programs worldwide, there is an increasing need for surrogate markers providing information to predict protection from re-infection or at least severe COVID-19 following natural infection and vaccination [29,30]. Therefore, we compared five serological assays not only with regard to dichotomized test accuracy but also analysed the degree of quantitative correlation with neutralizing capacity.

Regarding dichotomized results, the two COBAS assays proved superior to every other assay and delivered test results with a high accuracy. Therefore, both Roche assays can be proposed as a gold standard for dichotomous testing in a population-based setting. Our population-based results contradict the earlier findings by others reporting a sensitivity of 76% and non-determinable specificity for the COBAS_N based on convenience samples [31]. As their findings also strongly differ from the manufacturer’s information [32], one might attribute those results to the time point of blood drawal (e.g., shortly after infection) or cohort composition [31]. In contrast, all of our findings regarding sensitivity and specificity settle completely in line with the maufacturer’s data as well as larger meta-analyses attributing a general sensitivity of approximately 80 to 90% as well as a specificity of well over 95% to ELISA- and CLIA-based assays [4,26].

Comparing different tests to each other, we found similar concordance of our ELISA_G with the COBAS_N, whereas those previous findings also suggested high comparability of our ELISA_G with the commercially available Euroimmun test [5]. In contrast to others reporting lower agreement for the COBAS_S with neutralization [33], we found a high concordance of the COBAS_S with neutralization, in line with the manufacturers statement in the validation [34] and also in agreement with others comparing COBAS_S to ACE-2 Inhibition as a surrogate for neutralization [35]. This might be attributed, e.g., to higher sensitivity of our luciferase-reporter based neutralization assays as compared to a neutralization assay relying on a cytopathic effect as readout [33].

Regarding the quantitative effects of spectrum bias, we could show small to mid-range decreases in both sensitivity and specificity of all tests, when moving from our artificial, “test establishment setting” to a population-based setting, while the tests administered earlier recorded higher benefits in comparison to newer tests. This reflects the logical assumption that in the beginning of 2020 only limited access to positive sera was given [2], while later on manufacturers were able to utilize a more diverse pool of samples for determining assay performance [36].

That said, the impact of spectrum bias should definitely be considered in future population-based approaches, especially, when results are reliant on a single test. While in the past we applied latent class analysis comprising several different tests to determine the seroprevalence in our study population [10], we couldn’t show in this analysis any advancement of predicting serostatus from our LCA models as compared to the best single test in this study, thus rendering the LCA approach obsolete in this situation. Nonetheless, in the absence of a golden standard and without prior information on the actual and/or relative performance of different available tests, LCA can still provide a good opportunity to define epidemiological seroprevalence.

With international standardization of protective correlates on the horizon [30], quantitative readouts and the scope of their statement are gaining importance. Though only a surrogate, the SARS-CoV-2-VSV pseudovirus assay first introduced by Hoffmann et al. [14] as well as the lentiviral pseudotype system used by many others and us were found to correlate very well with real virus SARS-CoV-2 neutralization, while providing enhanced biosafety and throughput [15,16]. In contrast to the dichotomous assessment of the various tests with regard to their sensitivity and specificity, where the Roche COBAS_S as well as the Roche COBAS_N test behaved really well, both assays couldn’t provide a sufficient quantitative correlation to neutralization IC50s, and also the YHLO test didn’t reach satisfying correlation. Regarding the COBAS_S test, others found in agreement with this study a significant connection of dichotomous COBAS_S results with neutralization, but also in their hands the quantitative correlation between binding antibodies and virus neutralization proved to be not sufficiently good [33].

Overall, only the S-RBD-ELISA provided a satisfactory quantitative correlation, in the same ballpark as others found, with quite comparable assay formats such as the Euroimmun ELISA [3] or in house S-RBD-CLIA tests [37]. Consequently, the S-(RBD)-ELISA formats such as, e.g., our in-house assay or the commercially available Euroimmun- or Diasorin-tests are, amongst the analyzed tests, best suited to predict neutralization capacity and thus possibly for protection from infection or disease.

## 5. Conclusions

Whilst all used serological assays provide a solid test result for SARS-CoV-2 antibody seropositivity, the two Roche assays (ELECSYS anti-SARS-CoV-2 S&N) outperformed every other test in terms of identifying neutralizing sera. Regarding quantitative binding antibody readouts, only our in-house ELISA yielded satisfactory correlation to neutralization and thus qualified as possible predictive parameter of antibody-mediated protection. In terms of a potential spectrum bias during validation of assays with inpatient material we could show that a reconsideration of assay performance is necessary when moving from strictly defined clinical samples to a population-based setting, as otherwise estimates of specificity and sensitivity derived in such “test establishment settings” are biased upwards.

## Figures and Tables

**Table 1:** Applied antibody tests with manufacturer, test principle, test target, test strategy, antigen, cutoff, sensitivity and specificity as provided by the manufacturer.

Manufacturer	Principle	Target	Abbreviation	Antigen	Time after PCR	Sensitivity	Specificity	Reference
in house ELISA	ELISA	IgG; (IgA; IgM)	ELISA_G ELISA_A ELISA_M	Spike-RBD	>10 d	96% 92% 98%	99.3%	Peterhoff et al. 2021 [1]
Roche ELECSYS COBAS	ECLIA	total Ig	COBAS_S	Spike	≥14 d	99.5%	99.8%	IFU * [14]
Roche ELECSYS COBAS	ECLIA	total Ig	COBAS_N	Nucleoprotein	≥14 d	98.81%	99.98%	IFU * [15]
YHLO Biotech	ECLIA	total Ig	YHLO	Nucleo-protein & Spike	not specified	97.3%	96.3%	Wagner et al. 2021 [10]

* IFU: Manufacturer’s Instructions for Use.

**Table 2:** Agreement of dichotomous (seronegative vs. seropositive) test results within the complete group (*n* = 856 weighted to *n* = 4185) estimated by Cohen’s kappa (bold) with 95%-CIs.

Kappa 95% KI	COBAS_S	COBAS_N	ELISA_G	ELISA_A	ELISA_M	ELISA_ GAM	YHLO
COBAS_ S							
COBAS_N	**0.9646** 0.9467; 0.9825						
ELISA_ G	**0.9116** 0.8836; 0.9396	**0.9163** 0.8889; 0.9437					
ELISA_ A	**0.1916** 0.1395; 0.2438	**0.2097** 0.1560; 0.2634	**0.2114** 0.1559; 0.2669				
ELISA_ M	**0.1140** 0.0768; 0.1513	**0.1208** 0.0818; 0.1598	**0.1235** 0.0826; 0.1644	**0.1617** 0.0716; 0.2519			
ELISA_ GAM	**0.8488** 0.8129; 0.8847	**0.8579** 0.8230; 0.8928	**0.9262** 0.9006; 0.9519	**0.3216** 0.2707; 0.3725	**0.1298** 0.0928; 0.1668		
YHLO	**0.8853** 0.8537; 0.9170	**0.9146** 0.8868; 0.9423	**0.8741** 0.8405; 0.9076	**0.2402** 0.1823; 0.2981	**0.1225** 0.0794; 0.1657	**0.8116** 0.7719; 0.8513	

**Table 3:** Relationship between dichotomized antibody test results and dichotomized neutralization results. For each antibody test Cohen’s kappa, sensitivity, specificity, positive predictive values (PPV), negative predictive values (NPV) and accompanying 95%-CIs as well as the Youden-Index, are shown for the complete subgroup (*n* = 856 weighted to *n* = 4185) in the corresponding first row (labelled “all”) and for the reduced subgroup (*n* = 615 weighted to *n* = 4185) representing the test establishment setting (only participants with neutralization IC-50 < 1 or >100 included) in the second row labelled “Spectrum” (corresponding groups are highlighted by the same shadow).

Neutralization												
Test	Cohort	Kappa	95% CI	Sensitivity	95%-CI	Specificity	95% CI	Youden- Index J	PPV	95%-CI	NPV	95%-CI
COBAS _S	all	0.9719	0.9560; 0.9879	0.9719	0.9496; 0.9845	0.9971	0.9871; 0.9994	0.9690	0.9691	0.9009; 0.9909	0.9974	0.9906; 0.9993
	Spectrum	0.9961	0.9886; 1.0000	0.9954	0.9737; 0.9992	0.9997	0.9900; 1.0000	0.9951	0.9968	0.9261; 0.9999	0.9996	0.9924; 1.0000
COBAS _N	all	0.9418	0.9190; 0.9646	0.9386	0.9093, 0.9589	0.9966	0.9862; 0.9991	0.9352	0.9628	0.8904; 0.9880	0.9943	0.9861; 0.9976
	Spectrum	0.9986	0.9942; 1.0000	1.0000	0.9820; 1.0000	0.9990	0.9888; 0.9999	0.9990	0.9894	0.9143; 0.9988	1.0000	0.9932; 1.0000
YHLO	all	0.8619	0.8275; 0.8963	0.8526	0.8127, 0.8852	0.9944	0.9830; 0.9982	0.8470	0.9345	0.8487; 0.9732	0.9863	0.7189; 0.9924
	Spectrum	0.9614	0.9383; 0.9845	0.9587	0.9225; 0.9784	0.9952	0.9824; 0.9987	0.9539	0.9493	0.8546; 0.9835	0.9961	0.9866; 0.9989
ELISA _G	all	0.8915	0.8607; 0.9222	0.8917	0.8558; 0.9195	0.9894	0.9758; 0.9954	0.8811	0.8875	0.7953; 0.9412	0.9898	0.9800; 0.9949
	Spectrum	0.9734	0.9543; 0.9926	0.9817	0.9530; 0.9930	0.9914	0.9767; 0.9969	0.9731	0.9145	0.8129; 0.9634	0.9983	0.9900; 0.9997
ELISA _GAM	all	0.8736	0.8406; 0.9066	0.9224	0.8905; 0.9456	0.9500	0.9269; 0.9661	0.8724	0.6336	0.5391; 0.7189	0.9924	0.9832; 0.9966
	Spectrum	0.9206	0.8884; 0.9528	0.9908	0.9664; 0.9975	0.9495	0.9236; 0.9669	0.9403	0.6478	0.5390; 0.7431	0.9991	0.9912; 0.9999
*ELISA* _*M*	all	0.1092	0.0732; 0.1453	0.1043	0.0770; 0.1397	0.9930	0.9809; 0.9975	0.0973	0.5827	0.3287; 0.7994	0.9220	0.9020; 0.9383
	Spectrum	0.1993	0.1380; 0.2605	0.1651	0.1209; 0.2214	0.9944	0.9812; 0.9984	0.1595	0.7343	0.4510; 0.9029	0.9270	0.9035; 0.9452
*ELISA* _*A*	all	0.2315	0.1805; 0.2824	0.2445	0.2034; 0.2909	0.9667	0.9468; 0.9794	0.2112	0.4077	0.2756; 0.5545	0.9317	0.9123; 0.9471
	Spectrum	0.3326	0.2595; 0.4058	0.3211	0.2615; 0.3871	0.9627	0.9395; 0.9773	0.2838	0.4466	0.3006; 0.6024	0.9380	0.9153; 0.9549

**Table 4:** Agreement of seropositivity determined through various LCA-models and dichotomized neutralization results within the complete group (*n* = 856 weighted to *n* = 4185). Shown are: Tests included in the model, Cohen’s kappa, sensitivity, specificity and Youden-Index accompanied by 95%-CIs.

		Neutralization							
	Included in Model	Kappa	95% CI	Sensitivity	95% CI	Specificity	95% CI	Youden -Index	95% CI
LCA 1 *	ELISA_G; COBAS_N; YHLO	0.9220	0.8958; 0.9483	0.9152	0.8822; 0.9395	0.9976	0.9879; 0.9995	0.9128	0.8701; 0.9390
LCA 2	ELISA _GAM; COBAS_N; YHLO	0.9244	0.8985; 0.9503	0.9178	0.8852; 0.9417	0.9976	0.9879; 0.9995	0.9154	0.8731; 0.9412
LCA 3	ELISA_G; COBAS_N; YHLO; COBAS_S	0.9514	0.9305; 0.9722	0.9491	0.9216; 0.9672	0.9968	0.9866; 0.9993	0.9459	0.9082; 0.9665
LCA 4 **	ELISA _GAM; COBAS_N; YHLO; COBAS S	0.9514	0.9305; 0.9722	0.9491	0.9216; 0.9672	0.9968	0.9866; 0.9993	0.9459	0.9082; 0.9665

* Identical to model LCA 2b: ELISA_G, ELISA_A, ELISA_M, COBAS_N, and YHLO; ** Identical to model LCA 4b: ELISA_G, ELISA_A, ELISA_M, COBAS_N, COBAS S and YHLO.

## Data Availability

All authors declare that data and materials will be made available according to the guidelines of the journal.

## Supplementary Materials

The following are available online at https://www.mdpi.com/article/10.3390/diagnostics11101843/s1, Figure S1: Correlation of VSV pseudoviral versus SARS-CoV-2 neutralization, Figure S2: Model of positive and negative predictive values of each serologicial assay with respect to seroprevalence. Table S1: Spearmans rank-correlation coefficients of serological assays and neutralization.
