# Supplementary material for: Spectrum Bias and Individual Strengths of SARS-CoV-2 Serological Tests—A Population-Based Evaluation
Source: Diagnostics (Basel). 2021 Oct 6;11(10):1843. doi: 10.3390/diagnostics11101843 (PMC8534748; doi:10.3390/diagnostics11101843)
Supplement: Supplementary file 1 [file diagnostics-11-01843-s001.zip › diagnostics-1392615-supplementary.pdf]

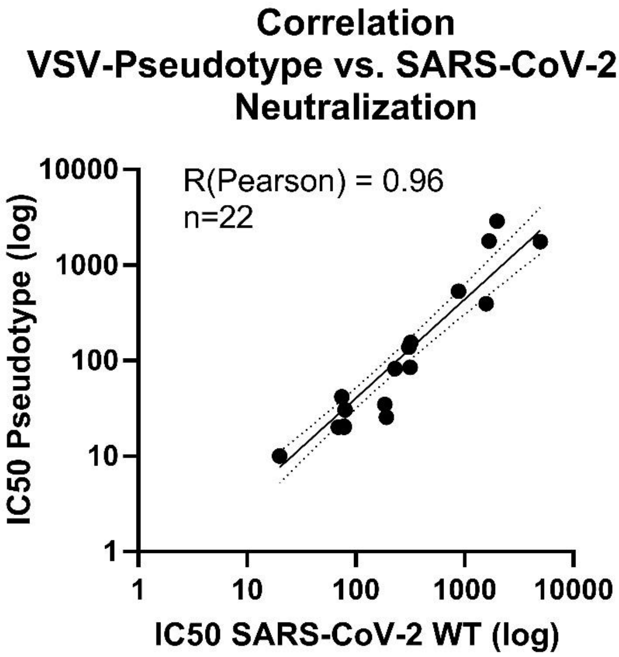

Supplementary Figure S1. Correlation of VSV pseudoviral versus SARS-CoV-2 neutralization (as described in (1)) for n = 22 sera (Pearson R=0.96).

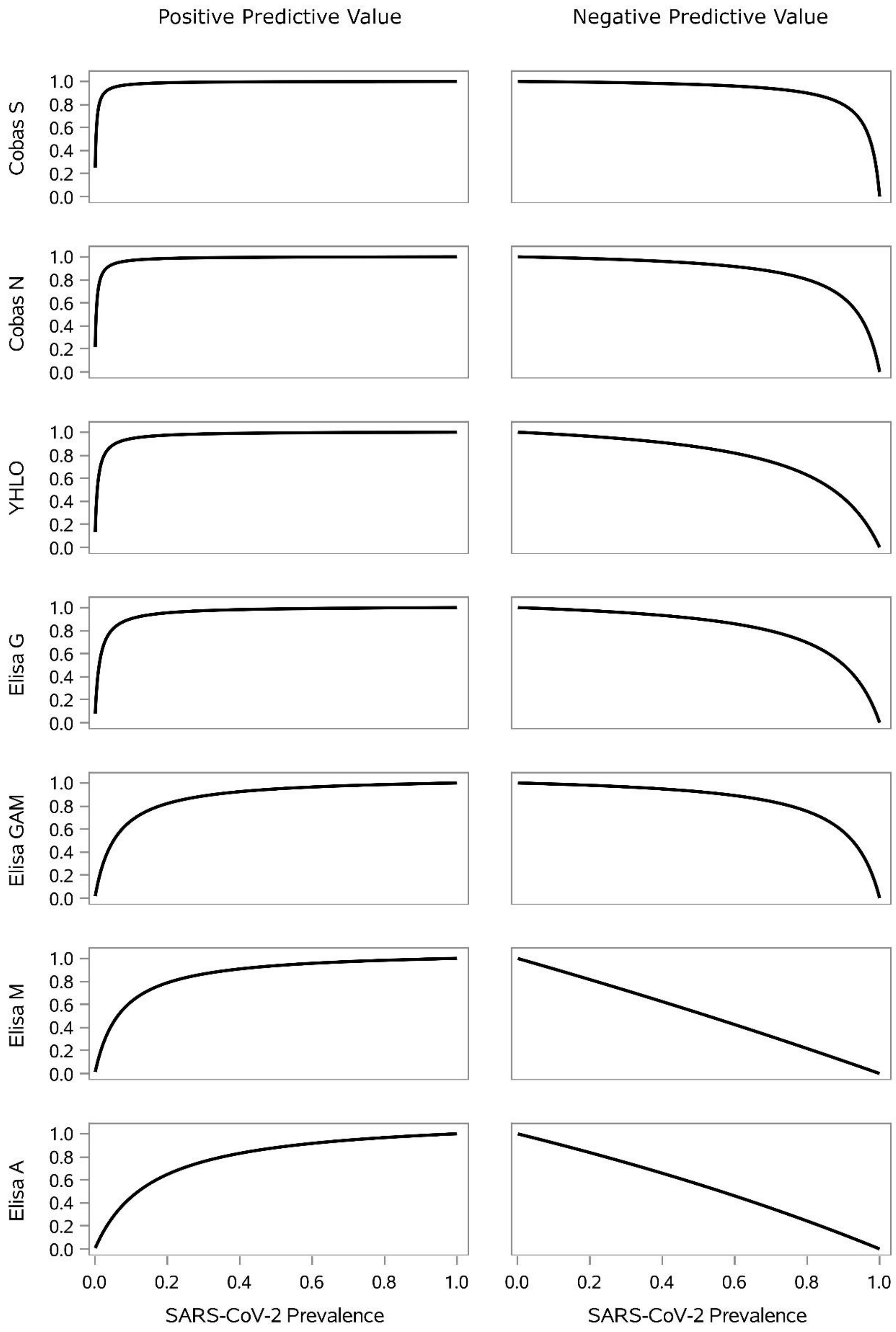

Supplementary Figure S2. Model of positive and negative predictive values of each serologicial assay with respect to seroprevalence.

Supplementary Table S1. Spearmans rank-correlation coefficients of serological assays and neutralization, within in all tests (ELISA_G, COBAS_N, COBA_S; YHLO; Neutralization) positive population (n = 310) to quantify general monotonous connection with 95% CI. (Further experimental inclusion constraints: COBAS_S ≤ 2500; ELISA_G ≤ 15; Neutralization IC_{50} ≤ 2560)

Spearman 95% CI	COBAS_S (log)	COBAS_N (log)	Elisa_G (lin)	YHLO (log)	Neutra-lization (log)
**COBAS_S (log)**					
**COBAS_N (log)**	**0.3667** 0.2692; 0.4641				
**Elisa_G (lin)**	**0.7714** 0.7136; 0.8291	**0.4256** 0.3316; 0.5196			
**YHLO (log)**	**0.4903** 0.4036; 0.5770	**0.7323** 0.6764; 0.7882	**0.5772** 0.4988; 0.6555		
**Neutra-lization (log)**	**0.5300** 0.4451; 0.6148	**0.3028** 0.2039; 0.4017	**0.6592** 0.5872; 0.7312	**0.4658** 0.3760; 0.5555	
